# Severe Lung Abscess and Sepsis Caused by *Klebsiella ozaenae* in a Malnourished Patient With Psychiatric Disease

**DOI:** 10.1002/rcr2.70482

**Published:** 2026-01-19

**Authors:** Tomoyuki Araya, Toshiyuki Kita, Tomoaki Yoneda, Takayuki Higashi, Ryo Hara, Hazuki Takato

**Affiliations:** ^1^ Department of Respiratory Medicine NHO Kanazawa Medical Center Kanazawa Ishikawa Japan

**Keywords:** *Klebsiella ozaenae*, lung abscess, malnutrition, psychiatric disease, sepsis

## Abstract

Severe infections caused by *Klebsiella ozaenae* are extremely rare, and their clinical characteristics remain poorly defined. We report a case of *Klebsiella ozaenae* sepsis with severe lung abscesses in a malnourished patient with psychiatric disease, requiring 3 months of antimicrobial therapy for complete resolution.

## Graphical abstract

Severe *Klebsiella ozaenae* infections are extremely rare. We report a malnourished patient with psychiatric disease who developed sepsis and lung abscesses requiring 3 months of antimicrobial therapy.

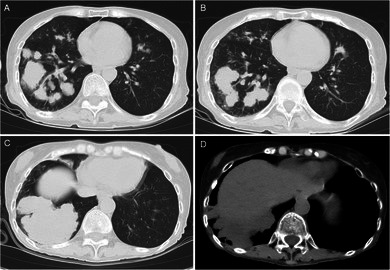

Severe infections caused by *Klebsiella ozaenae* are extremely rare, and their clinical characteristics remain poorly defined, with only limited reports of invasive disease [1, 2].

A 58‐year‐old woman with a history of eating disorder and major depressive disorder presented with fever and dyspnea. She had no history of diabetes, smoking or alcohol use. On admission, she presented with septic shock (temperature 39.3°C; blood pressure 68/50 mmHg), severe malnutrition (body mass index 14.6 kg/m^2^), and was mildly drowsy.

Chest computed tomography revealed multiple irregular, mildly lobulated nodules and mass‐like lesions with relatively low internal attenuation in both lungs, predominantly in the right lower lobe, without cavitation or pleural effusion (Figure 1). Laboratory tests showed leukopenia (1900/μL), markedly elevated C‐reactive protein (40.95 mg/dL), blood urea nitrogen of 44.2 mg/dL and serum creatinine of 2.43 mg/dL.

**FIGURE 1:**
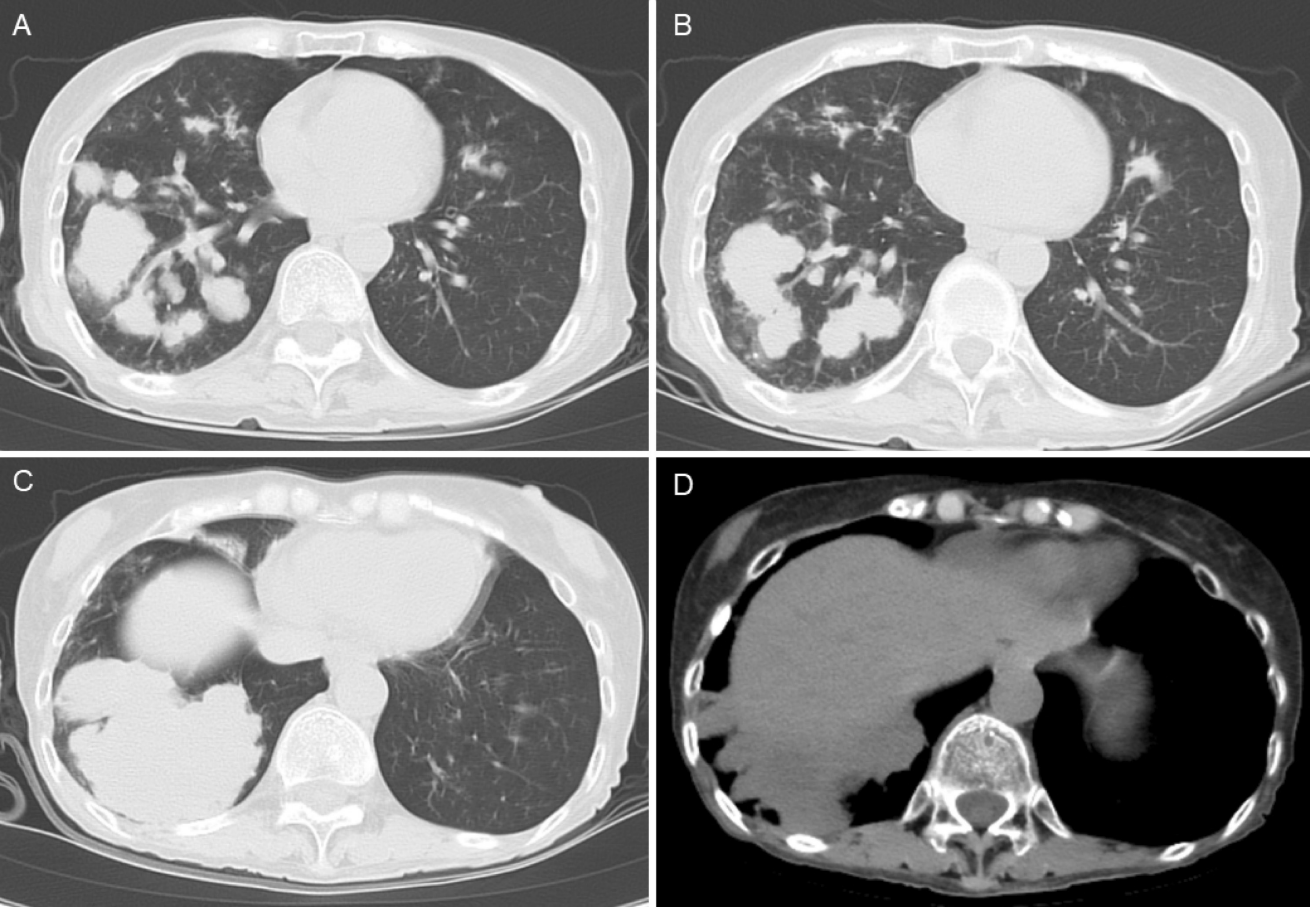
Chest computed tomography on admission demonstrating multiple irregular, mildly lobulated nodules and mass‐like lesions with relatively low internal attenuation in both lungs, predominantly in the right lower lobe, without cavitation or pleural effusion. (Panels A and B) show lobulated nodular lesions consistent with abscess formation. (Panel C) demonstrates a larger mass‐like abscess lesion. (Panel D) (mediastinal window) highlights the relatively low internal attenuation within the mass‐like abscess lesion.

Blood and sputum cultures grew *Klebsiella ozaenae* , leading to a diagnosis of sepsis with severe lung abscesses. Piperacillin–tazobactam was administered as empiric therapy because of the severity of the clinical presentation. Because clinical improvement was insufficient, after 3 weeks antimicrobial therapy was de‐escalated to ampicillin–sulbactam based on susceptibility results. However, severe diarrhoea developed, and therapy was subsequently switched to oral levofloxacin, to which the isolate was susceptible. A total of 3 months of antimicrobial therapy was required to achieve clinical cure. Serial chest radiographs demonstrating the clinical course are shown in Figure 2. The pulmonary lesions showed prolonged activity during the treatment course. Residual radiographic abnormalities persisted 3 months after clinical cure.

**FIGURE 2:**
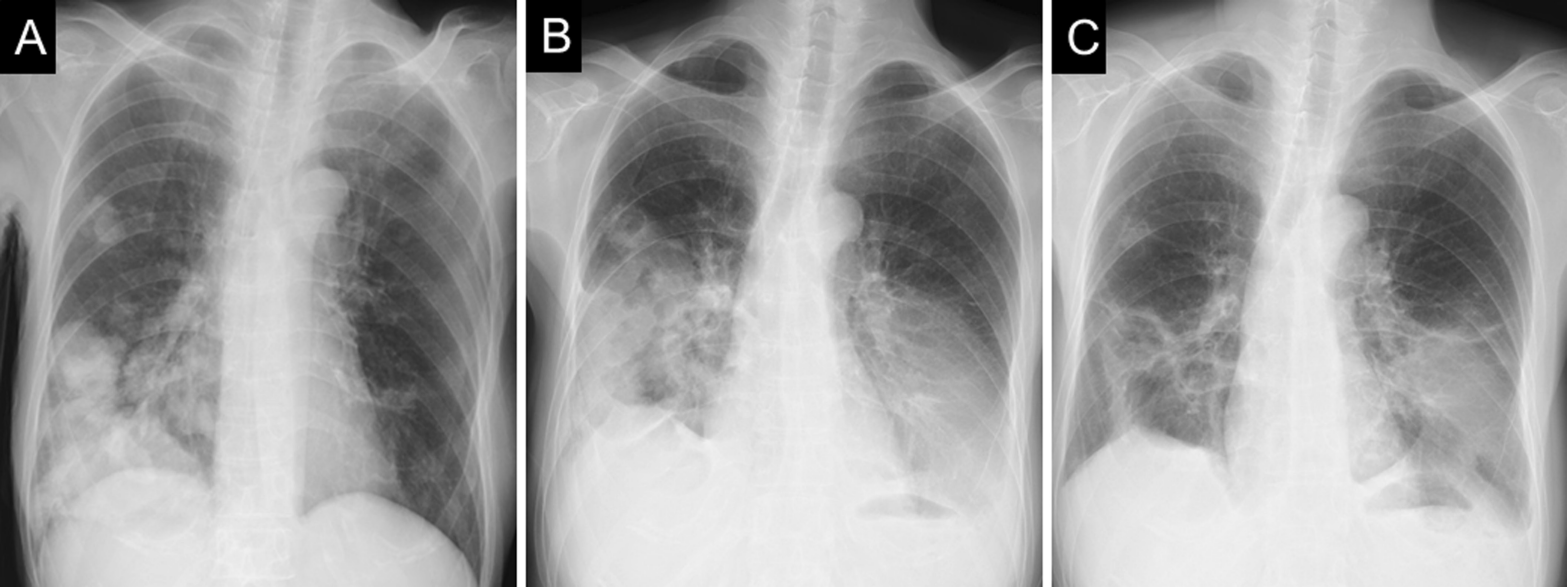
Serial chest radiographs illustrating the clinical course of severe lung abscesses caused by *Klebsiella ozaenae* . (A) Chest radiograph on admission showing multiple nodular and mass‐like opacities in both lung fields, predominantly in the right lower lung. (B) Chest radiograph 1 month after initiation of antimicrobial therapy demonstrating interval reduction in the size of multiple nodular and mass‐like lesions, with partial cavitation; however, worsening of the left lung abscess lesion is observed, along with newly developed bilateral pleural effusions. (C) Chest radiograph at 3 months, at the time of clinical cure, showing near‐complete resolution of bilateral pleural effusions; however, residual radiographic sequelae of bilateral lung abscesses persist.

This case suggests that although *Klebsiella ozaenae* rarely causes severe infections, even when initial symptoms are subtle, rapidly progressive severe infection in patients with poor general condition, such as malnourished patients with psychiatric disease, should prompt consideration of *Klebsiella ozaenae* as a potential causative pathogen when it is isolated from any clinical culture.

## Author Contributions

Tomoyuki Araya drafted the manuscript and verified the clinical data. Toshiyuki Kita supervised the work and critically reviewed the manuscript. Tomoaki Yoneda, Takayuki Higashi, Ryo Hara and Hazuki Takato reviewed the manuscript and contributed to data verification. All authors read and approved the final manuscript.

## Funding

The authors have nothing to report.

## Ethics Statement

The case report was approved by the ethics committee of the NHO Kanazawa Medical Center (approval no. R07‐057). The study was conducted ethically in accordance with the World Medical Association Declaration of Helsinki.

## Consent

The authors declare that written informed consent was obtained for the publication of this manuscript and accompanying images using the consent form provided by the Journal.

## Conflicts of Interest

The authors declare no conflicts of interest.

## Data Availability

The data that support the findings of this study are available on request from the corresponding author. The data are not publicly available due to privacy or ethical restrictions.
